# Predictors of optimal uptake of intermittent preventive treatment of malaria in pregnancy using sulfadoxine-pyrimethamine and outcome of pregnancy in selected health facilities: a cross-sectional study in Northern Ghana

**DOI:** 10.1186/s12936-023-04501-w

**Published:** 2023-03-06

**Authors:** Yaa Nyarko Agyeman, Bougangue Bassoumah, Joseph Owusu-Marfo

**Affiliations:** 1 Department of Population and Reproductive Health, School of Public Health, University for Development Studies, Tamale, Ghana; 2 Department of Epidemiology, Biostatistics and Disease Control, School of Public Health, University for Development Studies, Tamale, Ghana

## Abstract

**Background:**

Ghana adopted the 2012 World Health Organization (WHO) policy on intermittent preventive treatment of malaria in pregnancy (IPTp) and implemented it in 2014 in all regions of Ghana. Despite the implementation of this policy, there has been an unacceptably low percentage of eligible women receiving the optimal dose of IPTp in Ghana which leaves millions of pregnant women unprotected from malaria. The study, therefore, assessed the predictors of three or more doses (optimal dose) of sulfadoxine-pyrimethamine (SP) in Northern Ghana.

**Methods:**

A cross-sectional study was conducted among 1188 women in four selected health facilities in Northern Ghana from September 2016 to August 2017. Information on socio-demographic and obstetric characteristics reported SP use, and maternal and neonatal outcomes were collected which was double-checked from the maternal health book as well as the antenatal care register. Pearson chi-Square and ordered logistic regression were used to determine the predictors of reported optimal SP use.

**Results:**

Out of the 1146 women, 42.4% received 3 or more doses of IPTp-SP as recommended by the national malaria control strategy. SP uptake was significantly associated with antenatal care (ANC) attendance (aOR 0.49, 95% CI 0.36–0.66, P < 0.001), primary education (aOR 0.70, 95% CI 0.52–0.95, P = 0.022), four or more antenatal care visits (aOR 1.65, 95% CI 1.11–2.45, P = 0.014), ANC care visit in second trimester (aOR 0.63, 95% CI 0.49–0.80, P < 0.001) and third trimester (aOR 0.38, 95% CI 0.19–0.75, P = 0.006) and malaria infection during late gestation (aOR 0.56, 95% CI 0.43–0.73, p < 0.001).

**Conclusion:**

The percentage of pregnant women who received three or more doses is below the target of the National Malaria Control Programme (NMCP). The push factors for the optimal use of SP are higher educational attainment, four or more ANC visits, and early initiation of ANC. The study also confirmed earlier findings that IPTp-SP uptake of three or more doses prevents malaria in pregnancy and improves birth weight. The uptake of IPTp-SP among expectant women will be informed and increased by encouraging formal general education beyond the primary level and encouraging early initiation of ANC visits.

## Background

Pregnant women and their unborn babies are exposed to several harmful diseases including malaria which remains a global public health problem [1]. The most affected groups are pregnant women and children under 5 years of age. Most maternal deaths and miscarriages have been caused by malaria infection [2, 3].

It is estimated that about 125 million pregnancies in malaria*-*endemic areas of the world suffer yearly from malaria-related infant mortalities in the region of 75,000 and 200,000 [4]. In sub-Saharan Africa, the main infecting parasite is *Plasmodium falciparum,* which is responsible for 99% of all malaria in pregnancy (MiP) cases [5]. In areas where parasite transmission is stable, over 20 million pregnancies are at risk annually [6–8]. These mortalities are due to limitations in the effective detection and management of malaria cases in pregnant women and the further risk of anaemia in the mothers. Nationally, pregnant women who reported to out-patient departments (OPD) with malaria in 2014 were 224,542 (representing 2.7%, that is the proportion attributable to malaria). Out of this number, 6.7% were admitted because of MiP. The case fatality rate was 1.0%. At the regional level, the Northern Region recorded the highest number of deaths due to malaria [9]. Analyzed data in the country revealed that Ghana has the highest (48%) prevalence of MiP in the sub-region both in the wet (59%) and in the dry seasons (41%) [10].

One of the interventions for controlling the negative effect of malaria in pregnancy is the use of SP. Until 2012, IPTp-SP consisted of administering at least two doses of SP to pregnant women (at least once a month) spaced in time by at least a month. The WHO Evidence Review Group recommends the use of SP under Directly Observed Therapy (DOT) starting from the second trimester or after quickening and at subsequent visits separated by a month interval until delivery in October 2012 [11]. Through the National Malaria Control Programme (NMCP), Ghana adopted the implementation of the use of IPTp-SP in 2014 as part of the antenatal care package [12]. The increased dosing with three or more doses of SP was advocated to ensure maximum protection against malaria in pregnancy [13] and greater therapeutic results [14]. For instance, meta-analysis and mathematical models showed that taking at least three SP doses provided additional protection against malaria and was associated with low birth weight and stillbirth [13]. According to the Ghana Demographic Health Survey, there was an improvement in the coverage of two or more doses of IPTp-SP, ranging from 44% in 2008 to 68% in the two years preceding the 2014 survey. Further, the percentage of pregnant women who received 3 or more doses was 39% [15].

According to a 2014 survey conducted by the Ghana NMCP, SP coverage for IPT1, IPT2, and IPT3 was 54.1%, 38.7%, and 24.6%, respectively [16]. Moreover, women who received one or more doses of IPTp-SP increased from 58% in 2008 to 91% in 2019, while those who received two or more doses increased from 46 to 80%. During the same period, the percentage of women receiving three or more IPTp-SP doses increased from 28 to 61% [17]. Comparing the coverage of IPT over the past years, the 2019 Ghana Malaria Indicator Survey (GMIS) saw an improvement in the reported use of SP in Ghana. However, GMISs reported use of there or more doses of SP (61.0%) is less than the NMCP target of 85% set in 2011 [16]. This study is rationalized by the need to find out the predictors of the uptake of three or more doses of the SP to inform health policy planning, intervention, and implementation. The outcome of this research will contribute to the achievement of the Sustainable Development Goal (SDG) 3 and Target 3.3 [18].

## Methods

### Study design and setting

This research determined the predictors of optimal use of SP using a quantitative facility-based cross-sectional survey conducted in four selected health facilities within the Tamale metropolis catchment area in Ghana’s Northern Region. Teaching, Regional, and District health facilities within the Tamale Metropolis. Tamale Metropolis was chosen because it serves a diverse ethnic and socioeconomic population, and the Teaching Hospital also serves as a referral centre for the region. In Ghana, health care is organized at three levels: national, regional, and district [19]. The study drew on areas from Ghana's three levels of health care.

### Study population, sampling and sample size

The study population included post-delivery women who had delivered in any of the four health facilities in the Tamale metropolis. Women who attended ANC during pregnancy and had term delivery in any of the 4 selected health facilities were included in the study. Mothers who miscarried and had a preterm delivery, HIV positive and G6PD defects were excluded from the study. IPTp-SP is contraindicated in pregnant women in the first trimester of gestation [20].

The sample size was calculated based on the target population of pregnant women who attended ANC in 2015 (64,908) for the selected health facilities in the Tamale Metropolis. Using an estimated proportion of 85%, that is, the national target for IPTp coverage among pregnant women for 2011 in Ghana [21] at 95% confidence level and a precision of 3%, assuming a design effect of 2, the sample size obtained was calculated as 1080. The precision of 3% was assumed to help increase the sample size and decrease any uncertainty about the data.

A non-response rate of 10% was adjusted. The required sample size estimated was 1188. Probability Proportional to size (PPS) was used and the sample size for each health facility was 298, 352 and 538 for Teaching, Regional, and District health facilities, respectively. The sample size was 1188; however, 1146 respondents were used in the analysis because 46 of the pregnant women delivered before 36 weeks of pregnancy which made it impossible to assess the recommended number of doses expected.

### Study variables

The dependent variable was the receipt of three or more doses of IPTp 3, and the independent variables were socio-demographic factors, obstetric information, and maternal and neonatal outcomes. Table 1 shows independent variables and their definitions.

**Table 1:** Definition of independent variables

Variable	Definition
Age	Age in years, grouped as < 24, 25–29, 30–34. 35 +
Residence	Either urban, peri-urban or rural
Marital status	Either married or not
Educational level	Highest education attained, classified as none, primary, secondary, college/tertiary
Occupation	Grouped as farmers, artisan, salaried worker, trading, unemployed, others
Religion	Religious affiliation, categorized as Christians or Muslims
Ethnicity	Classified as Dagombas or others
Gravidae	Classified as primigravidae, secundigravidae, multigravidae
Parity	Number of live births, further grouped as nulliparous, primiparous, multiparous
No of ANC visits	Total number of ANC visits, grouped as 1–3 and 4 +
Trimester at 1st ANC	Gestation at the start of ANC, grouped as 1st 2nd and 3rd trimester
MiPs at 36 weeks	Presence of malaria parasites in the peripheral blood of pregnant women, categorized as either yes or no
Hb level at 36 weeks	Either Hb values less than 11 g/dl or Hb levels more than 11 g/dl, categorized as either yes or no
Delivery of LBW babies	Live-singleton babies who were weighed at delivery, either weight less than 2.5 kg or weight more than 2.5 kg
Delivery of stillbirth babies	Babies who are born dead after 37 complete weeks of gestation

### Data collection tool and method

The study questionnaire was designed to collect the following variables: socio-demographic and reproductive information, IPTp-SP uptake history, haemoglobin level, malaria in pregnancy, and birth outcomes. The study data were taken from three main sources: the maternal health record book, the birth register and through participant interviews. Data extracted from the maternal health record book were cross-checked with the register at the labour ward in accordance with the study protocol and the participants were subsequently interviewed directly to corroborate the validity of all obtained information.

During the interviews, the questionnaires were administered by the principal investigator and the study research assistants (who could also speak the local dialect) to the pregnant women. The respondents who could not understand and communicate in English language were interviewed by a research assistant in Dagbani, Hausa or Akan. The responses were then transcribed back to English and documented on the questionnaires. Data collection from the post-delivery women lasted for a maximum of about 10 min per woman. Data collection spanned a period of 12 months, from September 2016 to August 2017 to help account for seasonality in malaria.

### Quality control

Several precautionary measures were undertaken to enhance the quality of the study data. The study questionnaires were designed by using portions of UNICEF documents and extracts of published studies, so they reflected universal standards. A pilot study was done to ensure that the best methods were used to collect, process and examine study data and outcomes to avoid exaggerations.

Again, three dummy sessions were held for study data collectors to rehearse methods and communication strategies designed to extract the relevant information from interviewees. Data were edited daily on the field for necessary corrections by the principal investigator to minimize errors in the database. Two different data entry clerks were trained to enter the same data into the database to minimize errors and enhance data quality.

There were four scheduled data cleanings to ensure that all relevant information had been captured. Two different statisticians were made to analyze the study data to enhance the quality of the analysis. All collected study documents and data had restricted access, with access allowed for limited persons when necessary.

### Data analysis

All data entries and management were conducted using the Statistical Package for the Social Sciences software (SPSS) version 20.0 for Windows (SPSS Inc., Chicago) and transported to STATA 12.1 for the analysis (Stata Corporation, College Station, TX, USA). SP intake was grouped as no IPTp-SP, less than three (< 3) IPTp-SP doses and greater than or equal to three (≥ 3) IPTp-SP doses.

For the ordered logistic regression of predictors of optimal SP uptake, SP doses received were categorised into optimal (≥ 3) and suboptimal (< 3) based on WHO recommendations [22]. Pearson Chi-square test, and univariate and multivariate logistic regression analyses were performed. At the end of the bivariate analysis, variables for the multivariate analysis were selected into the model based on the p-value of 0.5 and the clinical significance of some risk factors [23, 24], results of past studies [25, 26] and interests in all the selected general characteristics irrespective of their statistical significance. P < 0.05 was considered statistically significant. The odd ratio (OR) was used to estimate the association between the dependent variable and the independent variables.

### Ethical consideration

The study is part of a large study titled “malaria control in pregnancy: an evaluation of the effectiveness of IPTp policy on maternal and neonatal health in the Tamale metropolis of Northern Ghana” which was approved by the Committee on Human Research, Publication and Ethics of Kwame Nkrumah University of Science and Technology/Komfo Anokye Teaching Hospital before the commencement of the study (CHRPE/AP/375/16). Informed consent was obtained from each respondent at the beginning of every individual interview. Confidentiality was maintained by the interviewers as records were coded and did not disclose the identity of respondents. The accessed data set were kept on a pass-worded computer.

## Results

### General characteristics and reported SP use among post-delivery women

Tables 2 and 3 show the general characteristics of the study respondents and the percentage use of IPTp-SP. Most of the post-delivery women attended ANC clinic at the District Hospital (45.1%), were below 24 years (34.2%) and resided in the urban communities (67.1%). Post-delivery women who had 4 or more antenatal care visits (89.5%) and attended ANC in their second trimester (53.6%) were in the majority. Most of the post-delivery women had anaemia (62.6%), however, 74.1% did not have malaria in pregnancy at 36 weeks of gestation. Post-delivery women who were between the ages of 25–29 (45.2%), had attained a higher level of education (53.4%), enrolled during the first trimester of their pregnancy (52.5%) and had four or more ANC visits (46.3%) reported higher usage of optimal dose of SP.

**Table 2:** General characteristics of reported SP use among post-delivery women

Characteristics	IPTp-SP Uptake			N 1146	Test statistic χ2 (Pearson chi2)	P-value	P-trend
	No SP use	< 3	≥ 3				
	232(20.2)	428(37.4)	486(42.4)				
Place of ANC attendance					89.3369	< 0.001	< 0.001
Teaching hospital	41(14.3)	96(33.3)	151(52.4)	288			
Regional hospital	63(18.5)	90(26.4)	188(55.1)	341			
District hospital	128(24.8)	242(46.8)	147(28.4)	517			
Age (years)					5.1113	0.530	0.472
< 24	82(20.9)	152(38.8)	158(40.3)	392			
25–29	78(21.0)	126(33.9)	168(45.1)	372			
30–34	49(20.4)	89(37.1)	102(42.5)	240			
35 +	23(16.2)	61(43.0)	58(40.8)	142			
Residence					28.7018	< 0.001	0.001
Urban	140(18.2)	270(35.1)	359(46.7)	769			
Peri-urban	45(20.3)	87(39.4)	89(40.3)	221			
Rural	47(30.1)	71(45.5)	38(24.4)	156			
Marital status					2.5934	0.273	0.145
No	20(23.5)	36(42.4)	29(34.1)	85			
Yes	212(20.0)	392(36.9)	457(43.1)	1061			
Educational level					24.0770	0.001	0.004
No school	121(21.7)	217(38.8)	221(39.5)	559			
Primary	59(23.9)	97(39.3)	91(36.8)	247			
Secondary	20(11.3)	70(39.5)	87(49.2)	177			
College/tertiary	32(19.6)	44(27.0)	87(53.4)	163			
Occupation					33.7751	< 0.001	0.187
Farmer	8(19.5)	23(56.1)	10(24.4)	41			
Artisan	39(17.6)	91(41.0)	92(41.4)	222			
Salaried worker	27(18.2)	39(26.4)	82(55.4)	148			
Trading	108(21.5)	173(34.5)	221(44.0)	502			
Unemployed	40(24.2)	76(46.1)	49(29.7)	165			
Others	10(14.7)	26(38.2)	32(47.1)	68			
Religion					7.1614	0.028	0.023
Muslim	218(21.4)	377(36.9)	426(41.7)	1021			
Christian	14(11.2)	51(40.8)	60(48.0)	125			
Ethnicity					0.2427	0.886	0.663
Dagomba	180(20.4)	332(37.6)	371(42.0)	883			
Others	52(19.8)	96(36.5)	115(43.7)	263			

*ANC* antenatal care*; MiP* malaria in pregnancy*; Hb* haemoglobin

**Table 3:** Characteristics of reported SP use among post-delivery women

Characteristics	IPTp-SP Uptake			N 1146	Test statistic χ2 (Pearson chi2)	P-value	P-trend
	No SP use	< 3	≥ 3				
	232(20.2)	428(37.4)	486(42.4)				
Gravidae					8.7019	0.069	0.565
Primigravidae	78(22.6)	133(38.6)	134(38.8)	345			
Secundigravidae	47(18.1)	83(32.1)	129(49.8)	259			
Multigravidae	107(20.0)	212(39.1)	223(41.1)	542			
Number of ANC visit					61.6319	< 0.001	< 0.001
1–3	42((35.0)	67(55.8)	11(9.2)	120			
4 +	189(18.5)	361(35.2)	474(46.3)	1026			
Trimester at 1st ANC							
1st trimester	65(13.1)	170(34.3)	260(52.5)	495	56.5247	< 0.001	< 0.001
2nd trimester	135(24.9)	238(38.8)	223(36.3)	614			
3rd trimester	14(37.8)	20(54.1)	3(8.1)	37			
MiP at 36 weeks					37.8570	< 0.001	< 0.001
No	149(17.5)	296(34.9)	404(47.6)	849			
Yes	83(28.0)	132(44.4)	82(27.6)	297			
Hb at 36 weeks					28.2421	< 0.001	< 0.001
Non-anaemic	63(14.7)	143(33.3)	223(52.0)	429			
Anaemic	169(23.6)	285(39.7)	263(36.7)	717			
Parity							
Nulliparous	84(21.9)	148(38.5)	152(39.6)	384	8.6770	0.70	0.80
Primiparous	44(17.7)	79(31.9)	125(50.4)	248			
Multiparous	104(20.2)	201(39.1)	209(40.7)	514			

*ANC* antenatal care*; MiP* malaria in pregnancy*; Hb* haemoglobin

Place of residence influenced the uptake of SP (p < 0.001). Pregnant women who resided in urban residences had the highest percentage of using the optimal dose during pregnancy (46.7%). Pregnant women who had no malaria infection (47.6%) were found to have used the optimal dose compared to those who had malaria infection (27.6%). There was a higher uptake of the optimal dose of SP among pregnant women who were not anaemic (52.0%) compared to those who were anaemic (36.7%).

### Proportion of post-delivery women with reported use of optimal doses of SP

**Fig. 1:**
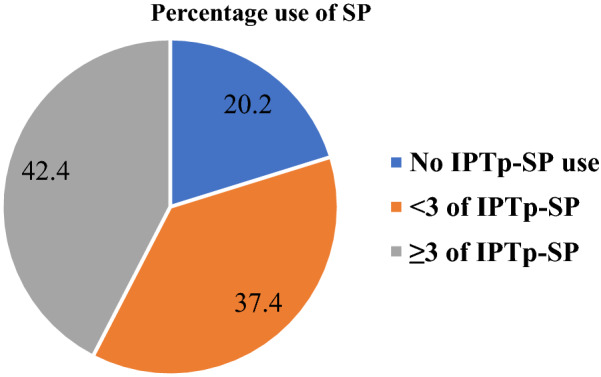
Stratification of SP doses

**Fig. 2:**
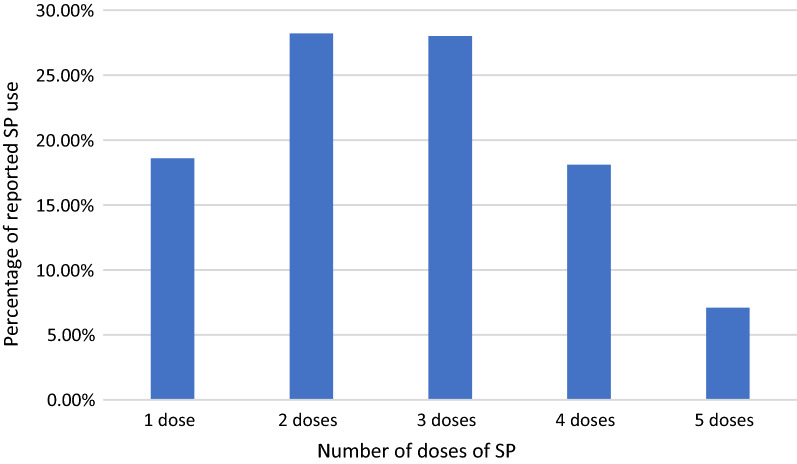
Number of doses received by post-delivery

Figure 1 represents the stratification of SP based on the doses. Three or more (**≥ **3**)** doses are considered the optimal dose recommended by the WHO. It could be observed that 42.4% of the post-delivery women reported usage of the optimal dose (Fig. 1) with 20.2% who did not take SP during pregnancy. Among those who reported uptake of SP during pregnancy, a higher proportion of the post-delivery women reported uptake of 2 doses (28.2%) with only 7.1% who reported uptake of 5 doses (Fig. 2).

### Ordered logistic model for the determinants of optimal SP usage

Tables 4 and 5 are the multivariable ordered logistic regression determining the factors that influenced the reported use of SP among post-delivery women. Women who attended ANC at the District Hospitals were 51% less likely (aOR 0.49 (0.36–0.66), p < 0.001) to take the optimal dose of SP compared to women who attended ANC at the Teaching Hospital. One of the important determinants for the reported use of SP is the number of times the woman attends ANC. Post-delivery women who had 4 or more antenatal care visits were more likely to use 3 or more doses of SP (aOR 1.65(1.11–2.45), p = 0.014) compared to those with less than 4 ANC visits. Post-delivery women who reported and enrolled in the antenatal care services in their second trimester were 37% less likely (aOR 0.63 (0.49–0.80), p < 0.001) to take the optimal doses compared to those who reported in their first trimester of gestation. From the same table, it was observed that post-delivery women who had malaria during their pregnancy were less likely (aOR 0.56(0.43–0.73), p < 0.001) to have taken the optimal dose compared to those who had no malaria infection in pregnancy. This could explain why probably they got infected. Post-delivery women who gave birth to low-birth-weight babies were 53% less likely (aOR 0.47(0.34–0.64), p < 0.001) to take the optimal dose of SP. This means that post-delivery women who reported usage of SP had protection against malaria infection and low birth weight delivery.

**Table 4:** Multivariable analysis for demographic factors influencing the uptake of optimal doses of SP

Characteristics SP 3 or more	Univariate regression		Multivariable regression	P-value
	cOR95% CI	P-value	aOR(95% CI)	
Hospitals				
Teaching Hospital	**Ref**		**Ref**	
Regional Hospital	1.0(0.75–1.37)	0.940	1.10(0.80–1.52)	0.552
District Hospital	0.39(0.29–0.51)	< 0.001	0.49(0.36–0.66)	< 0.001
Residence				
Peri-urban	Ref		Ref	
Urban	1.25(0.95–1.65)	0.115	1.15(0.86–1.55)	0.339
Rural	0.54(0.37–0.79)	0.001	0.73(0.49–1.08)	0.118
Educational level				
No school	Ref		Ref	
Primary	0.89(0.67–1.17)	0.401	0.70(0.52–0.95)	0.022
Secondary	1.60(1.16–2.19)	0.004	1.08(0.75–1.57)	0.677
College/tertiary	1.57(1.12- 2.20)	0.009	0.68(0.37–1.27)	0.230
Occupation				
Farmer	Ref		Ref	
Artisan	1.58(0.88–2.84)	0.126	0.81(0.43–1.53)	0.514
Salaried worker	2.38(1.28–4.44)	0.006	0.95(0.40–2.28)	0.917
Trading	1.57(0.90–2.75)	0.114	0.81(0.44–1.49	0.501
Unemployed	1.02(0.56–1.86)	0.947	0.62(0.32–1.20)	0.153
Others	1.96(0.98–3.92)	0.059	0.76(0.34–1.72)	0.509
Religion				
Muslim	Ref		Ref	
Christian	1.44(1.02–2.04)	0.037	1.00(0.69–1.48)	0.961

*cOR* crude odd ratio*, aOR* adjusted odd ratio*, 95%CI* 95% confident inferential

*p* < *0.05 considered statistically significant*

**Table 5:** Multivariable analysis for obstetric characteristics and birth outcomes influencing the uptake of optimal doses of SP

Characteristics SP 3 or more	Univariate regression		Multivariable regression	P-value
	cOR95% CI	P-value	aOR(95% CI)	
Gravidae				
Primigravidae	Ref		Ref	
Secundigravidae	1.50(1.10–2.03)	0.010	0.76(0.40–1.45)	0.400
Multigravidae	1.13(0.88–1.45)	0.344	0.71(0.29–1.71)	0.443
Parity				
Nulliparous	Ref		Ref	
Primiparous	1.48(1.09–2.00)	0.011	1.62(0.85–3.08)	0.146
Multiparous	1.06(0.83–1.36)	0.616	1.25(0.52–3.02)	0.616
Number of ANC visit				
1–3	Ref		Ref	
4 +	3.50(2.49- 4.93)	< 0.001	1.65(1.11–2.45)	0.014
Trimester at 1st ANC				
1st trimester	Ref		Ref	
2nd trimester	0.49(0.39–0.62)	< 0.001	0.63(0.49–0.80)	< 0.001
3rd trimester	0.20(0.11–0.36)	< 0.001	0.38(0.19–0.75)	0.006
Malaria in pregnancy at 36 weeks of gestation				
No	Ref		Ref	
Yes	0.47(0.37–0.60)	< 0.001	0.56(0.43–0.73)	< 0.001
Anaemia in pregnancy at 36 weeks of gestation				
No	Ref		Ref	
Yes	0.54(0.43–0.68)	< 0.001	0.84(0.65–1.20)	0.201
Delivery of LBW babies				
No	Ref		Ref	
Yes	0.36(0.27–0.47)	< 0.001	0.47(0.34–0.64)	< 0.001
Delivery stillbirth babies				
No	Ref		Ref	
Yes	0.50(0.35–0.72)	< 0.001	0.87(0.57–1.31)	0.503

*cOR* crude odd ratio*, aOR* adjusted odd ratio*, 95%CI* 95% confident inferential

*p* < *0.05* considered statistically significant

## Discussion

Though the reported use of SP is expected to prevent malaria in pregnancy and ameliorate the effects on maternal and neonatal health [15, 17], some studies have reported a high prevalence of MiP in some parts of Ghana [27–32].

In the current study, pregnant women residents in urban areas were the majority (46.7%) in terms of reported uptake of 3 or more doses of IPTp-SP. This is consistent with other studies which found that urban women are more likely to take three or more doses of SP [33, 34]. However, according to the GMIS women in rural areas (62.0%) were more likely to receive at least three doses, while urban women were more likely not to take SP more than one or two times. The difference could be due to study methodologies: GMIS sampled respondents from across the country, whereas this study’s sample was drawn from a metropolitan area in Ghana’s northern region. There was regional variation in the reported use of three or more doses of SP; Upper West was the highest performing region (78.0%), followed by Upper East (77.4%), Northern (64.5%), and Eastern (42.2%), which is similar to the findings of this study (42.4%) [17]. This suggests that ongoing campaigns to improve malaria prevention methods for expectant mothers should focus especially on rural areas.

Maternal education (college/tertiary; 53.4%) and being resident in the urban (46.7%) and peri-urban (40.3%) were impactful in higher uptake of IPTp-SP. This is consistent with findings from previous studies [34–37]. Education helps women to read literature, as well as listen to the radio and watch TV to understand the effects of malaria in pregnancy. It also helps people be inquisitive and to be open to new ideas [36, 38, 39] which in turn builds women's knowledge of health issues which helps them to make well-informed healthcare choices during pregnancy [40]. Educated women are better subscribers of malarial interventions and often complete their IPTp-SP dosage course [41, 42]. Studies conducted in Yendi and Chereponi in the Northern and the North-East Regions observed that women without formal education had difficulty in understanding health information during clinical counselling due to the language barrier and format of the information. Hence, they are unable to translate health information into practice [43]. Similarly, less educated women might come from poorer families and live in homes that lack electricity which makes it difficult to access the media easily [44, 45]. This might be the reason for the higher uptake compared to women without education. Facility-based health information should be based on needs assessment to effect relative change in women’s behaviour towards maternity care.

In this study, 42.4% of participants used the optimal dose (three or more doses), with 28.0%, 18.1%, and 7.1% using 3 doses, 4 doses, and 5 doses, respectively. The percentage of pregnant women who received three or more doses of SP is similar to previous studies [34, 39, 46, 47] but lower than the findings of a study conducted in southern Ghana (71.0%) [26] and the 2019 GMIS findings (61.0%) [48]. However, the percentage recorded in this study is an improvement over the percentage (39.0%) reported by the 2014 Ghana Demographic and Health Survey [15] and the NMCP (24.6%) in 2014. Pregnant women who took 3 or more doses of IPTp-SP increased significantly between 2014 (39.0%) and 2019 (61.0%) but remained unchanged between 2016 (60.0%) and 2019 (61.0%) [48]. The proportion of post-delivery women who used IPTp-SP 4 and 5 were 18.1% and 7.1%, respectively, which were higher than the national rates of 4.2% and 1.2%, respectively [16]. The low uptake of IPT 4 and 5 might be due to the late reporting for ANC services in Ghana as studies show that irrespective of the nationwide progress in ANC coverage, timing and number of visits remain a maternal health concern [15, 49, 50]. This observation is further sustained by the results from Table 5 which found that early initiation of ANC and 4 or more ANC (aOR 1.65(1.11–2.45), p = 0.014) visits increased the likelihood of taking three or more doses of SP which corroborates the findings of a study done in Malawi and confirms the WHO updated guidance on ANC[46].

Most of the post-delivery women visited ANC at least four times (89.5%), however, less than half (46.3%) of women reported uptake of three or more doses of SP which is consistent with the findings of a study conducted in Kumasi [35], Gushegu [52], Ghana and Tanzania [53, 54].

In Table 5, pregnant women who initiated their first ANC during the second (aOR 0.63 (0.49–0.80), p < 0.001) and third trimester (aOR 0.38 (0.19–0.75), p = 0.006) were less likely to take three or more doses of SP compared to those who initiated it during the first trimester. This re-affirms the need to place more emphasis on early ANC initiation [9, 21, 34, 49, 50].

The NMCP should step up efforts to promote early ANC initiation and raise awareness about the importance of IPTp-SP and other malaria prevention measures. If they understand the benefits of IPTp, they will seek it out or request it at ANC [34], resulting in optimal IPTp-SP uptake [54]. The updated WHO ANC recommendations stress on early promotion of ANC interaction at either the facility or community level [51, 55].

It was discovered that pregnant women who had malaria at 36 weeks of gestation [aOR 0.56(0.43–0.73), p < 0.001] and gave birth to low-birth-weight babies [aOR 0.47(0.34–0.64), p < 0.001] were less likely to have taken the optimal dose than those who had no malaria in pregnancy and gave birth to normal weight babies. These findings support the argument that using an optimal dose of SP is still effective in preventing malaria in pregnancy [14, 27] and improving birth weight [13]. As a result, the NMCP and the Ghana Health Service, Ghana, should implement interventions aimed at increasing the uptake of at least three doses of SP.

## Limitations

The study used a cross-sectional study design, which has the disadvantage of making it impossible to infer causation. Again, there was the possibility of the women reporting the usage of IPTp inaccurately. However, these women were interviewed while they were still on admission and the information given was double-checked from their maternal health care records as well as the ANC register book at the various facilities.

## Conclusion

According to the findings of the study, early initiation of ANC and four or more ANC visits during pregnancy increased the uptake of the optimal dose of SP. Postpartum women who had late gestational malaria and had low birth weight babies reported low SP uptake. Women with a primary education who were enrolled in ANC during the second and third trimesters reported less use of SP as an intermittent malaria preventive treatment in Northern Ghana. This study emphasizes the importance of expanding general education beyond primary school and encouraging early ANC visits to increase coverage of the recommended IPTp-SP uptake among pregnant women, thereby preventing gestational malaria infection and improving birth weight.

## Data Availability

The datasets used and/or analyzed during the current study are available from the corresponding author on reasonable request.

## Abbreviations

ANC: Antenatal care
aOR: Adjusted odd ratio
DOT: Directly observed therapy
GMIS: Ghana Malaria Indicator Survey
IPTp: Intermittent preventive treatment of malaria in pregnancy
Mip: Malaria in pregnancy
NMCP: National Malaria Control Programme
OPD: Out-patient departments
OR: Odd ratio
PPS: Probability proportional to size
SDG: Sustainable development Goal
SP: Sulfadoxine-pyrimethamine
UNICEF: United Nations Children’s Fund
WHO: World Health Organization
